# TB treatment outcomes among TB-HIV co-infections in Karnataka, India: how do these compare with non-HIV tuberculosis outcomes in the province?

**DOI:** 10.1186/1471-2458-13-838

**Published:** 2013-09-11

**Authors:** Suresh Shastri, Balaji Naik, Anita Shet, Bharat Rewari, Ayesha De Costa

**Affiliations:** 1 Karnataka State AIDS Prevention Society, Bangalore, Karnataka, India; 2 Revised National TB Control Program, Bangalore, Karnataka, India; 3 St. Johns National Academy of Health Sciences, Bangalore, Karnataka, India; 4 Division of Global Health, Karolinska Institutet, Stockholm, Sweden; 5 National AIDS Control Program, New Delhi, India

**Keywords:** HIV TB co-infection India program epidemiology treatment outcomes

## Abstract

**Background:**

India accounts for 23% of the global incidence of TB cases; it also has an estimated 2.3 million HIV infections. Of the 2 million TB incident cases, 5% occurred in HIV infected persons. The country has large national TB and HIV control programs. This paper describes characteristics of TB-HIV co-infection cases registered under the program in Karnataka province, India. Treatment outcomes for coinfected patients are compared with those for TB patients in the province.

**Methods:**

Program reports from the National AIDS Control program and the National TB control program for Karnataka province (a high HIV prevalence state, population 61 million) were analysed. Data from patients registered in each program in 2010–2011 was studied.

**Results:**

Of the 6,480 adult co-infections, a third occurred in women; 78% of patients were initiated on ART. Among the cohort 73% had pulmonary TB, and 46% reported sputum positivity for acid fast bacilli. Treatment success among co-infected patients not on ART (54%) were significantly lower compared to those already on ART (80%); death and default rates were higher in the non-ART group. Treatment success proportions (75%) for the co-infected patients were similar to those for the 51,966 patients registered under the TB program. Death rates among co-infected patients (15%) were twice as high as for TB patients under the program, though default and failure rates were lower.

**Conclusion:**

Co-infected patients already on ART demonstrated better TB outcomes in than those not on ART. Compared to those with TB only, co-infected patients had similar TB treatment success rates and lower rates of treatment default and failure. Integration of TB-HIV collaborative activities will strengthen our battle to control TB and HIV globally.

## Background

The convergence of the tuberculosis (TB) and the HIV epidemics pose new public health challenges [1]. The interaction between HIV and TB in co-infected persons is bidirectional and synergistic; on one hand, HIV-1 infection predisposes to the development of active TB, and, on the other, the course of HIV-related immunodeficiency is worsened by active TB infection [2]. TB is the most common opportunistic infection seen in HIV patients as well as a leading cause of death in these patients. The lifetime risk of TB in immune-competent persons is 5-10% whereas in an HIV-infected person, the annual risk of TB is 5-15% [3].

India has the highest TB burden in the world and accounts for 23% of the global incidence of TB cases i.e. 2 million of the 8.8 million incident cases in 2011 were from India [4]. A recent paper estimated that nearly 5% of the 2 million TB incident cases were HIV seropositive [5]. While the HIV epidemic in the country is showing a declining trend with a 56% drop in the number of new infections from 1996 levels [6], there are still an estimated 2.27 million people living with HIV infection [7]. India has well-structured and functional national programs for the control of both HIV and TB; the National AIDS Control Program (NACP) and the Revised National TB Control Program (RNTCP) respectively. The country has established cross-referral mechanisms in most provinces, where NACP has implemented intensified TB case finding in its HIV counseling and testing centres [8]. As TB is the most common opportunistic infection among the HIV-infected, HIV care providers screen clients for TB signs and symptoms and refer TB suspects to the nearest microscopy centre or diagnostic facility for further TB examinations/tests. Despite having a relatively well functioning national program in place, there are few published reports on HIV-TB co-infections from India.

This paper reports on demographic and clinical characteristics, and short-term treatment outcomes of HIV-TB co-infected patients registered in the HIV program in Karnataka, one of the highest HIV prevalence provinces of India. Further, TB treatment outcomes in these co-infected patients are compared with those among non-HIV-infected TB patients registered under the National TB Control Program within the same province.

## Methods

### Study design and study setting

**Figure 1:**
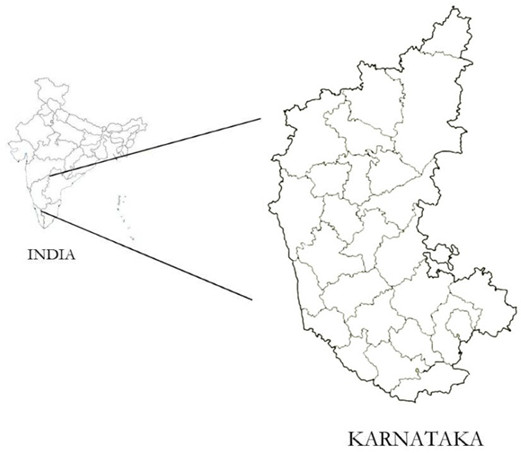
Karnataka province, India.

This was a retrospective cross-sectional study using routine program data from Karnataka province in South India. Karnataka (Figure 1), has a population of 61.1 million spread across 30 districts. Karnataka is one of four large south Indian states; it experiences a relatively advanced HIV epidemic, with the adult HIV prevalence in several districts exceeding 1% for the past 9 years. It has an estimated 0.25 million infections. In 2007, the prevalence among antenatal women was estimated at 0.9% [9]. The National AIDS Control Organisation of the government of India through its provincial wing, the Karnataka State AIDS Prevention Society, provides anti-retroviral treatment (ART) and follow up throughout the province through 40 district and sub-district ART centers, besides 123 link-ART centres (these are centers further in the hinterland which serve as points where patients living in these areas can collect their medication to reduce travel time and opportunity costs for patients). In addition the state also runs 4 centers through collaborations with reputed private health institutions in the province. Provinces in the country follow a strategy of provider-initiated HIV testing and counseling for all patients registered for TB treatment as recommended by WHO and UNAIDS. While TB treatment is initiated under the TB program at a treatment center close to the patients residence, the TB treatment and patient response is also monitored by the ART center physician in case of co-infected patients. Records of all co-infected patients being followed up at these centers from 1 April 2010 to 31 March 2011 were analysed.

### ART for HIV-infected TB patients

During the study period, the national guidelines for ART for HIV TB co-infection followed the 2006 World Health Organization Guidelines for ART. As per these guidelines, all HIV-infected persons with pulmonary TB, extra-pulmonary or disseminated TB with a CD4-lymphocyte count ≤350/mm^3^ were considered eligible for ART. The most common ART regimen initiated was zidovudine or stavudine plus lamivudine plus efavirenz.

### TB patients in the national TB control program

In the province, tuberculosis control program services are available through a decentralized network of primary health care facilities which provide general health services including diagnosis and treatment for TB. All TB patients are treated with standardized fully intermittent thrice weekly short-course regimens (6–9 months) administered under direct observation and are registered at one of the 125 sub-district level TB program management units according to Indian program guidelines [10]. Data for treatment outcomes for non-HIV-infected TB patients in the same province under the RNTCP program was obtained for patients registered between Apr 1 2010 and Dec 31 2010.

### Data collection

For HIV and TB co-infected patients, reports from each center were obtained as excel spreadsheets which were verified and collated. Every ART center has in its registers the HIV infected patients registered at the respective center who are on TB treatment. This information is filled in by staff at the respective ART centers. These staff provide reports on co infected patients to the provincial level ART program office, from where the spreadsheets of data on co-infected patients were obtained.

Data on age, sex, last (most recent) CD4 count, category of TB treatment, ART status and TB treatment outcomes were extracted from the records. Program outcomes were defined as per the WHO Stop TB program. The following TB treatment outcomes were considered: cured, treatment completed, died, failure or defaulted.

Similarly TB program data from the DOTS program across the province flows in from the peripheral DOTS centers to the provincial program office. The reports (unlike the coinfected patient reports) are summary statistics of patients categories by outcome, and so not contain individual characteristics of each patient.

Definitions of treatment outcomes: The national TB control program defines: (1) “Cured” as a patient who was initially sputum smear positive, has completed treatment and had negative sputum smears on two occasions, one of which is at the end of the treatment. If at the end of treatment, sputum smear is not done, the patient is classified as “treatment completed”. In case of smear negative or extrapulmonary TB, a patient who has received full course of treatment and has not become smear positive at the end of the treatment is also refered to as treatment completed. The total of “cured” and “treatment completed” is taken as “success”. (2) “Failure” refers to any TB patient who is smear positive at five months or more after starting the treatment and (3) “Defaulted” is any patient who has interrupted treatment consecutively for more than 2 months [11].

Data analysis: Analysis was carried out in Predictive Analytics Software, version 20 (PASW v 20). Descriptive analysis was performed and results were expressed as means, medians and simple proportions. Tests of significance for differences between proportions were carried out. Results were expressed as odds ratio and 95% CI.

### Ethical approval

Ethical approval for this study was obtained from the Institutional ethical review board of St. John’s National Academy of Health Sciences, Bangalore, India.

## Results

A total of 6,480 adult HIV-TB co-infected patients were registered at the ART centers in the province during the one-year study period, 35.6% of whom were women. The mean age of the patients was 37.7 years and median recent CD4 count was 143 cells/mm^3^, similar in both men and women. First-line ART was had already been initiated in 78% of patients diagnosed with TB. Baseline characteristics of patients not on ART and on ART were similar (mean age 38.1 and 37.5 years respectively; 63.8% and 84.2% males respectively). Nearly three-quarters (73.2%; 4,741/6,480) of all co-infected patients had pulmonary TB. Of these, 46% were sputum positive for acid fast bacilli. The remaining 26.8% had extra-pulmonary TB. Extra-pulmonary TB was seen in a higher proportion of patients who had initiated ART (28.5%) than those who had not as yet done so (14.2%) (OR = 2.4, 95% CI 2.03-2.84). The proportions of pulmonary and extra-pulmonary TB were similar among men and women, as were the proportions of smear positive TB. A majority of the patients (87%) were new TB infections while 9.6% were those who had either defaulted treatment, received incomplete treatment, or those who had relapsed.

### Outcomes of TB treatment in HIV and TB co-infected patients

Of the 6,008 co-infected patients for whom data on TB treatment outcomes were available, 929 were still on treatment. Of the remaining 5079 patients, three quarters (3,776 or 74.5%) had completed treatment successfully (i.e. cured or treatment completed). A total of 296 patients (5.8%) had defaulted, while 22 had failed treatment. There were 797 deaths (15.7%), with similar mortality proportions in the pulmonary and extra pulmonary TB infection groups. Forty patients had not been initiated on TB treatment because they refused treatment or wished to be treated for their TB in the private sector. TB treatment outcomes were significantly better for those patients who had already initiated ART prior to their diagnosis of TB: This compared to those who had not yet initiated ART, had a higher proportion of treatment completions/cures, lower rates of death, default or failure as shown in Table 1.

**Table 1:** A comparison of TB treatment outcomes in HIV-TB co-infected patients diagnosed with TB taking and not taking ART

***Treatment outcome***	***Not on ART (n = 1024) N (%)***	***On ART (n = 4016) N (%)***	***OR (95% CI)***
**Treatment success**	552 (54)	3191 (79.5)	1.47 (1.39-1.56)
**Death**	256 (25)	541 (13.5)	0.53 (0.47-0.61)
**Default**	105 (10.3)	118 (3)	0.28 (0.22-0.36)
**Failure**	10 (1)	13 (0.3)	0.33 (0.14-0.75)

### Outcomes in TB patients in the RNTCP program compared with those for HIV TB co-infected patients

Treatment successes were similar among co-infected TB patients compared to those with only TB within the National TB program (OR = 0.9, 95% CI = 0.88-0.92). Death rates were expectedly higher in the co-infection group (OR = 2.22, 95% CI = 2.03-2.38). However rates of default and treatment failure were higher among TB patients in the TB program as shown in Table 2.

**Table 2:** Comparison of treatment outcomes in patients with HIV TB co-infection and TB in Karnataka

***Treatment outcome***	HIV TB coinfection^*^(n = 5079) N (%)	***TB only (n = 51966)***^***#***^***N (%)***	***OR (95% CI)***
**Treatment success**	3,776 (74.3)	34,924 (79.9)	0.90 (0.88-0.92)
**Death**	797 (15.7)	3,670 (7.1)	2.22 (2.03-2.38)
**Default**	226 (4.4)	4,557 (8.8)	0.50 (0.44-0.58)
**Failure**	23 (0.5)	1,249 (2.4)	0.18 (0.12-0.28)

*Represents data from 1 Apr 2010 to 31 Mar 2011.

^#^Represents data from 1 Apr 2010 to 31 Dec 2011.

## Discussion

This study constitutes the first report of TB treatment outcomes in a large HIV-TB co-infection cohort in India. Globally and in India, TB is one of the most common opportunistic infections affecting people with HIV. This assumes importance in a country like India which has 2.7 million HIV infections and 23% of the world’s incident TB cases. HIV infection is often cited as an important reason for failure to control TB, and for causing a resurgence in TB worldwide. While this is true, our results suggest that implementation of program guidelines in a coordinated manner can result in good treatment outcomes among those co-infected with TB and HIV.

In this study, pulmonary involvement occurred in about 73% of all HIV infected patients with TB. This is in line with previous reports from India [12]. Smaller studies from Thailand have reported a lower proportion of pulmonary TB (60%) [13] while a report from Kenya [14] stated 87% co-infected patients had pulmonary TB. While overall treatment success rates in our co-infected cohort were close to 75%, they were significantly better in the group that had been already initiated on ART compared to those who were not on ART. Similarly, mortality rates were twice as high in the group that had not started ART at the time of TB treatment. Recent studies from different countries have repeatedly shown that early initiation of ART among co-infected individuals even during the intensive phase of anti-tuberculous therapy can decrease mortality among those with HIV/TB co-infection [15-18]. While from a biological point of view, patients on ART have lower viral replication and hence better host responses, from a program point of view, these patients are regularly clinically followed at the ART centers where adherence is repeatedly emphasized, possibly contributing to better outcomes in this group. This finding in our Indian cohort lends local contextual support to the recently introduced (Nov 2011) Indian program guideline of initiating ART in all HIV-TB co-infected individuals, as soon as possible in the intensive phase of anti-tuberculous treatment, regardless of CD4 counts [19]. A recent study from the same province in India has shown that only 21% of a cohort of co-infected patients was on ART at the time of TB diagnosis [20]. The same report suggests that despite operational weaknesses in the program, ART could be extended to all HIV-infected TB patients irrespective of CD4 count with relatively little additional burden on the national HIV control program [20].

Treatment success proportions in the co-infected cohort were similar to those achieved within the routine TB control program in the province. There was an expectedly significantly higher death rate among the co-infected patients (16% versus 7%) in the province. Globally, TB-associated mortality in co-infected patients is three times higher than mortality among TB only patients. There are a number of possible explanations that have been proposed for the increased mortality among co-infected patients. The location and extent of TB is influenced by the degree of immunosuppression, often increasing the difficulty of diagnosis and hence delaying treatment initiation, resulting in higher mortality [21]. Immunological studies have also shown the host responses to *M. tuberculosis* enhance HIV replication [22,23], thus accelerating the natural progression of HIV and further depressing cellular immunity. Decreased gut absorption of anti-tuberculous drugs has been suggested by some reports [24], leading to impaired treatment outcomes including death. Non-initiation or delayed initiation of ART in this cohort has also contributed to higher mortality [25]. Our analysis also indicated that rates of default and failure were significantly higher among TB only patients than in the TB HIV co-infected cohort. This is possibly because of a significant proportion of re-treatment patients entering the TB program (20% of all registered patients) compared to the co-infected cohort. In addition it is likely that the emphasis on multidisciplinary care and adherence support to patients within the ART program may play a role in contributing towards the lower rates of default and failure among the HIV/TB co-infected group, particularly among those on ART.

This study is limited by the non-inclusion of the patients taking ART privately. Only co-infected cases within the ART program in the province are considered in this study, hence the number of and outcomes for such cases not registered in the ART program cannot be ascertained. However this excluded group is likely to be small. The same is also the case for patients receiving TB treatment outside the program. The exact time duration between initiation of ART and ATT cannot be identified reliably from this dataset. Another limitation of the data set is the absence of information on sites of extra pulmonary infection. Bias is a problem as co-infected patients are included in the main TB program registers (co-infected patients are not removed from the registers and reported separately), though in the summary statistical reports from the TB program used in this study, they are not identifiable as such. The strength of this study lies in the large size of the co-infected cohort, and the results are likely to represent the majority of those accessing HIV or TB care in the country.

## Conclusion

In summary, we have shown that TB treatment success rates in Karnataka among the HIV/TB co-infected population were similar to those with TB only accessing care within the national TB control program. Moreover, co-infected patients demonstrated lower rates of treatment default and failure compared to those with TB only, suggesting that the national ART guidelines with emphasis on adherence and counseling support may have a role in these positive outcomes. The benefit of ART on TB outcomes in also clearly demonstrated in these results and supports the WHO 2010 recommendation on the early initiation of ART in co-infected cases. Ongoing efforts to integrate TB-HIV collaborative activities will strengthen the program further and will accelerate the march towards TB and HIV control both nationally and globally.

## Competing interests

The authors declared that they have no competing interests.

## Authors’ contributions

SS; AS and ADC designed the study and worked with drafting the manuscript. BN worked with data and analysis from the TB program and drafting the manuscript. BR participated in study design and manuscript drafting and reviewing. All authors read and approved the final manuscript.

## Pre-publication history

The pre-publication history for this paper can be accessed here:

http://www.biomedcentral.com/1471-2458/13/838/prepub
